# Gastroesophageal Reflux Burden Before and After Exercise Challenge in Children with Poorly Controlled Asthma: A 48-Hour MII-pH Study

**DOI:** 10.3390/arm94050066

**Published:** 2026-09-16

**Authors:** Urszula Jedynak-Wąsowicz, Izabela Jastrzębska, Małgorzata Sładek, Liliana Klim, Ewa Cichocka-Jarosz, Grzegorz Lis

**Affiliations:** 1 Department of Pediatrics, Children’s University Hospital, Jagiellonian University Medical College, 31-008 Krakow, Poland; ewa.cichocka-jarosz@uj.edu.pl (E.C.-J.);; 2 Department of Internal Medicine and Gerontology, Jagiellonian University Medical College, 31-008 Krakow, Poland; 3 Department of Gastroenterology and Nutrition, Children’s University Hospital, Jagiellonian University Medical College, 31-008 Krakow, Poland; 4 Students’ Scientific Group at Children’s Diseases Clinic, Children’s University Hospital, Jagiellonian University Medical College, 31-008 Krakow, Poland; liliana.klim@student.uj.edu.pl

**Keywords:** asthma, exercise challenge test, GER, multichannel intraluminal impedance, physical activity, overweight, obesity

## Abstract

**Highlights:**

**What are the main findings?**

- The absolute number of reflux episodes was higher after ECT, but a rate-based sensitivity analysis in participants with available analyzable recording times did not confirm a higher reflux frequency.
- Exploratory analyses suggested an association between excess body weight and greater acid exposure; these findings did not remain significant after correction for multiple testing.

**What are the implications of the main findings?**

- No statistically significant association between abnormal acid exposure and exercise-induced bronchoconstriction was demonstrated in this small exploratory cohort.
- Excess body weight predisposes to higher acid exposure and may influence asthma control and GER status, especially in the absence of typical reflux symptoms.

**Abstract:**

**Background**: Gastroesophageal reflux and exercise-induced bronchoconstriction (EIB) commonly coexist with asthma, but the effect of exercise on reflux remains unclear. We aimed to assess the prevalence of abnormal impedance-detected acid exposure in children with partially controlled or uncontrolled asthma, its association with EIB, and changes in reflux parameters after an exercise challenge test (ECT). **Methods**: Thirty patients aged 7–18 years underwent spirometry, treadmill ECT, and 48 h multichannel intraluminal impedance-pH monitoring. EIB was defined as a ≥10% fall in FEV_1_, and abnormal acid exposure as impedance-detected acid reflux lasting ≥1.2% of recording time. **Results**: Abnormal acid exposure was identified in 14/30 patients (46.7%), whereas EIB occurred in 6/30 (20.0%), with no significant association between them. The absolute number of reflux episodes was higher after ECT in the count-based analysis (43.0 [35.2–69.8] vs. 33.5 [21.2–54.5], *p* = 0.006); however, this finding was not confirmed after normalization to analyzable recording time in a sensitivity analysis. Overweight or obesity was present in 9 patients (30.0%) and correlated positively with acid exposure. BMI-for-age z-score correlated positively with acid exposure (ρ = 0.41, *p* = 0.025), but these findings did not remain significant after correction for multiple testing. **Conclusions**: Using the prespecified acid-exposure criterion, abnormal acid exposure was frequently identified in this cohort. No statistically significant association with EIB was demonstrated; however, the small number of EIB events limits definitive conclusions. The higher absolute post-ECT reflux episode count was not confirmed in a rate-based sensitivity analysis, and a causal effect of exercise on reflux cannot be established.

## 1. Introduction

Childhood asthma is one of the most common chronic diseases worldwide, and remains a major global health concern [1]. In Poland, the prevalence of asthma among children aged 7–10 years has been estimated at up to 10.4% [2], although a substantial proportion of cases may remain undiagnosed [3]. Exercise-induced bronchoconstriction (EIB) is common in patients with asthma and appears to occur particularly frequently in children [4,5]. It is thought to result primarily from airway cooling and water loss during increased ventilation, leading to osmotic and vascular changes and the release of bronchoconstrictive mediators [6,7].

Despite the risk of EIB, children with asthma are encouraged to participate in regular physical activity. Appropriately selected exercise improves physical fitness and quality of life and may contribute to favorable clinical outcomes [8,9,10]. However, strenuous physical activity may also provoke symptoms arising from conditions that coexist with asthma and potentially complicate the interpretation of exercise-related respiratory complaints [11].

Gastroesophageal reflux (GER) is common in patients with asthma, although the direction and clinical significance of this association remain uncertain [12]. GER-related respiratory manifestations have been proposed to result from direct microaspiration of gastric contents or indirectly through vagally mediated reflex pathways triggered by stimulation of acid-sensitive esophageal receptors [13,14,15]. In children with asthma, the reported prevalence of GER varies widely, from 19.3% to 80.0%, depending on patient selection and the diagnostic method used [16]. Controlled studies have reported GER more frequently in children with asthma than in healthy controls [16], and gastroesophageal reflux disease (GERD) is considered a potentially relevant comorbidity in patients whose asthma remains poorly controlled despite appropriate treatment [14,17].

The relationship between physical activity and GER is also complex. Although regular physical activity may be associated with a lower long-term risk of GERD [18], gastrointestinal and reflux-related symptoms are frequently reported during strenuous exercise, particularly in endurance sports [11,19]. Vigorous exercise may reduce gastrointestinal blood flow, impair gastric emptying and esophageal motor function, and compromise esophagogastric junction competence [15,20,21,22]. These mechanisms provide a potential biological basis for changes in reflux burden associated with intensive physical activity.

Obesity represents an additional clinically relevant comorbidity of both asthma and GER [23]. Increased intra-abdominal pressure and alterations in esophagogastric junction function may promote reflux in individuals with excess body weight [24], whereas obesity-related mechanical and inflammatory effects may increase respiratory symptoms and complicate assessment of asthma control [25]. The coexistence of asthma, reflux, and excess body weight may therefore be particularly relevant in pediatric patients.

Only a few studies have evaluated the relationship between GER and EIB, and the available findings remain inconclusive [26,27,28]. Moreover, data obtained using combined multichannel intraluminal impedance and pH monitoring (MII-pH) in children with asthma undergoing a standardized exercise challenge are scarce. Unlike conventional pH monitoring, MII-pH detects reflux episodes independently of their acidity and provides additional information on bolus movement and esophageal clearance.

Asthma medications have also been proposed as potential modifiers of GER. Beta-2 adrenergic agonists and other sympathomimetic agents may decrease lower esophageal sphincter pressure and alter esophageal motility [29,30]. However, the clinical relevance of these mechanisms in children receiving regular inhaled asthma treatment remains uncertain.

The primary objective of this study was to assess the prevalence of abnormal acid exposure on 48 h MII-pH monitoring in children with partially controlled or uncontrolled asthma and to examine its association with EIB. We also compared MII-pH parameters during the recording segments before and after a standardized exercise challenge test (ECT). Secondary exploratory objectives included assessment of the associations between asthma medication use, nutritional status, and reflux parameters.

We hypothesized that abnormal acid exposure would be common in children with inadequately controlled asthma and might be associated with EIB. We further hypothesized that reflux burden would differ between the recording segments preceding and following ECT.

## 2. Materials and Methods

### 2.1. Study Design and Ethical Approval

This prospective observational study was conducted at the Allergy Outpatient Clinic and the Department of Pediatrics of Jagiellonian University Medical College. The present investigation was an exploratory substudy nested within a larger prospective study examining gastroesophageal reflux and its treatment in patients with asthma. Clinical procedures and prospective data collection relevant to the present analysis were performed between 2007 and 2011.

The prospective parent study and clinical data collection were approved by the Bioethics Committee of Jagiellonian University Medical College on 28 June 2007 (approval no. KBET/68/L/2007). Written informed consent was obtained from the parents or legal guardians of all patients. Children provided assent when appropriate for their age.

### 2.2. Study Population

The study included 30 consecutive pediatric patients aged 7–18 years with partially controlled or uncontrolled asthma. No separate a priori sample-size calculation was performed for this exploratory substudy. The analytic sample comprised 30 children from the parent study who had the assessments required for the present analysis, including MII-pH monitoring and ECT.

Asthma diagnosis, control and treatment step were determined according to the Global Initiative for Asthma (GINA) criteria applicable at the onset of the study period [31]. Asthma control was additionally assessed using the standard Asthma Control Test (ACT; score range 5–25), which was administered uniformly to all participants. A score of ≥20 was considered indicative of controlled asthma [32].

Medical records were reviewed for respiratory and gastrointestinal symptoms, including wheezing, cough, abdominal pain, chest pain or heartburn, vomiting or regurgitation, and throat clearing. Information regarding sensitization to indoor and outdoor allergens, concomitant allergic rhinitis, and regular asthma treatment was collected.

The exclusion criteria were:

- acid-suppressive medication or antibiotic therapy within 4 weeks before the enrollment;
- typical symptoms of GER;
- asthma exacerbation associated with a respiratory tract infection;
- asthma exacerbation associated with seasonal allergen exposure.

The study used a within-subject design for the temporal analysis of reflux, with each participant serving as his or her own control during the continuous MII-pH recording before and after ECT.

### 2.3. Study Protocol

The study protocol included baseline spirometry, 48 h MII-pH monitoring, and a standardized ECT. MII-pH monitoring was continuous, with the catheter remaining in place throughout the exercise challenge and subsequent spirometric assessments.

The actual time of the exercise challenge was used as the temporal cut-off separating the pre-ECT and post-ECT recording segments. Because the ECT did not necessarily occur at the exact midpoint of the recording, the analyzable duration of the two segments could differ. Meal periods and other non-analyzable intervals were excluded.

As a sensitivity analysis, reflux episode counts were normalized to analyzable recording time and expressed as episodes per hour in participants for whom segment-specific analyzable durations were available.

Participants were encouraged to maintain their usual diet, sleep schedule, and daily activities, except during the standardized exercise challenge. Regular asthma treatment was continued throughout the study, including on the day of ECT.

This pragmatic study design was intended to assess reflux and bronchial responses under conditions resembling patients’ everyday life and usual asthma treatment rather than after medication withdrawal.

Patients and their caregivers completed a two-day diary documenting mealtimes, changes in body position, respiratory symptoms, gastrointestinal symptoms, and other relevant events. Meal periods were excluded from the MII-pH analysis.

### 2.4. Exercise Challenge Test

The ECT was performed according to the ATS recommendations applicable during the study period [33].

Baseline spirometry was performed immediately before exercise using a Lungtest 1000 spirometer (MES, Krakow, Poland). Heart rate and peripheral oxygen saturation were monitored throughout the test using pulse oximetry.

The ECT was performed 2 h after a hospital-provided breakfast adjusted to the patient’s age, sex, and individual nutritional requirements.

Only children who were able to cooperate adequately and whose baseline FEV_1_ was at least 80% of the predicted value underwent ECT.

The exercise challenge was conducted on a treadmill. Treadmill speed and inclination were progressively adjusted during 6 min of running to achieve 80–90% of the age-predicted maximal heart rate during the final 4 min. The age-predicted maximal heart rate was calculated as 220 minus age in years.

Patients wore a nose clip during exercise. Ambient temperature was maintained at 19–22 °C and relative humidity was below 55%.

Spirometry was repeated 5, 10, and 20 min after completion of the exercise challenge. EIB was diagnosed when the greatest decrease in FEV_1_ recorded at any of these time points was at least 10% relative to the pre-exercise value.

The percentage decrease in FEV_1_ was calculated as:

ΔFEV_1_ (%) = [(FEV_1_ pre-exercise − lowest post-exercise FEV_1_)/FEV_1_ pre-exercise] × 100.

### 2.5. Multichannel Intraluminal Impedance–pH Monitoring

All participants underwent 48 h MII-pH monitoring using the Sleuth System (Sandhill Scientific, Highlands Ranch, CO, USA).

After fasting for at least 4–6 h, a soft 2.3 mm polyvinyl impedance catheter equipped with six impedance channels and one pH sensor (ZPI-BS-46 ComforTec 6Z/1pH; Sandhill Scientific) was introduced transnasally into the esophagus under local anesthesia.

The distal pH sensor was positioned 3–5 cm above the lower esophageal sphincter. The estimated insertion depth was determined according to Strobel’s formula [34]. Correct probe positioning was confirmed by chest radiography, with the distal pH sensor positioned approximately two vertebral bodies above the diaphragm [35].

Patients were encouraged to maintain their usual activities and report respiratory and gastroesophageal symptoms using the event marker and study diary.

### 2.6. Analysis of MII-pH Recordings

The recordings were initially analyzed automatically using dedicated Sandhill Scientific software (BioVIEW Analysis 5.0.9) and were subsequently reviewed manually by a single investigator. When interpretation was uncertain, a second experienced investigator was consulted.

Investigators analyzing the MII-pH tracings were blinded to participants’ clinical characteristics and ECT results.

The examination protocol was based on published MII-pH methodology and pediatric recommendations. Pediatric reference values used for interpretation were derived from Mousa et al. [36].

The MII-pH parameters evaluated included:

- acid exposure percentage time;
- non-acid exposure percentage time;
- total reflux exposure percentage time;
- mean acid clearance time;
- duration of the longest reflux episode; and
- total number of liquid and mixed reflux episodes.

Parameters were analyzed for the complete recording and separately for the pre-ECT and post-ECT 24 h periods.

Non-acid exposure percentage time was defined as the proportion of analyzable monitoring time during which impedance-detected reflux episodes were associated with an intraesophageal pH of at least 4.

Total reflux exposure percentage time represented the proportion of analyzable monitoring time associated with all impedance-detected reflux episodes.

Mean acid clearance time was defined as the mean duration of episodes during which esophageal pH remained below 4.

The total number of reflux episodes included liquid and mixed reflux episodes detected by impedance monitoring.

### 2.7. Definition of Abnormal Acid Exposure

The percentage time of impedance-detected acid reflux was defined as the proportion of analyzable monitoring time during which an impedance-detected reflux episode was associated with distal esophageal pH < 4. A value of ≥1.2% for the complete recording was used as the prespecified classification threshold. This value corresponds to the 90th percentile for total impedance-detected acid reflux exposure reported by Mousa et al. [36] in children aged >12 months. Because these values were derived from clinically referred children rather than a strictly asymptomatic healthy population, they were regarded as pediatric reference values rather than definitive normative limits. A sensitivity analysis was additionally performed using the corresponding 95th-percentile threshold of ≥1.3%. Patients meeting this predefined criterion were classified as MII-pH-positive, whereas those with values below this threshold were classified as MII-pH-negative.

Other MII-pH parameters, including non-acid and total reflux exposure percentage time and the number of liquid and mixed reflux episodes, were analyzed as measures of reflux burden but were not used to define MII-pH status.

Three patients had abnormal non-acid reflux parameters. Because this subgroup was too small for meaningful separate statistical analysis, non-acid reflux status was not used to define the primary study groups.

### 2.8. Assessment of Nutritional Status

In a post hoc exploratory analysis, nutritional status was assessed using age- and sex-specific BMI (body mass index)-for-age z-scores based on the 2007 World Health Organization growth reference for children and adolescents aged 5–19 years [37].

Body mass index was calculated as weight in kilograms divided by height in meters squared. Overweight was defined as a BMI-for-age z-score greater than +1 standard deviation and obesity as a BMI-for-age z-score greater than +2 standard deviations. Because of the small sample size, participants with overweight or obesity were combined into a single excess-weight group.

Associations between nutritional status, MII-pH status, acid exposure percentage time, and the number of reflux episodes were assessed as exploratory analyses.

### 2.9. Symptom Association Analysis

The symptom index (SI) was defined as the percentage of recorded symptoms temporally associated with a reflux episode. An SI above 50% was considered positive.

Symptom association probability (SAP) was used to estimate the probability that the observed association between reflux episodes and symptoms was not attributable to chance [38]. An SAP above 95% was considered positive.

Symptom association analyses included belching and respiratory symptoms such as cough, wheezing, and dyspnea.

### 2.10. Statistical Analysis

Statistical analyses were performed using R software, version 3.0 (R Foundation for Statistical Computing, Vienna, Austria).

Categorical variables are presented as numbers and percentages. Continuous variables are presented as medians with interquartile ranges (IQRs), unless otherwise specified.

Because continuous variables did not follow a normal distribution, comparisons between two independent groups were performed using the Mann–Whitney U test. Paired comparisons between the pre-ECT and post-ECT recording periods were performed using the Wilcoxon signed-rank test.

Categorical variables were compared using the chi-square test or Fisher’s exact test when expected cell counts were small.

Post hoc exploratory analyses of nutritional status included Fisher’s exact test for the association between excess weight and MII-pH status and Spearman’s rank correlation analysis for associations between BMI-for-age z-score and continuous reflux parameters.

The association between MII-pH status and EIB was considered the primary analysis. Secondary and exploratory analyses were considered hypothesis-generating. The Benjamini–Hochberg false discovery rate procedure was applied to the retained secondary and exploratory inferential tests. Nominal *p* values are reported in the main tables, with adjusted q values provided in Supplementary Table S1.

All statistical tests were two-sided. A *p* value below 0.05 was considered statistically significant.

Given the small sample size and exploratory nature of the study, subgroup analyses and secondary analyses were considered hypothesis-generating.

## 3. Results

### 3.1. Study Population

The study included 30 consecutive children aged 7–18 years with partially controlled asthma (n = 4; 13.3%) or uncontrolled asthma (n = 26; 86.7%). The cohort included 18 boys and 12 girls, and the mean age was 12.7 ± 3.3 years.

Clinical characteristics of the study cohort, GINA treatment step and ACT score at baseline are shown in Table 1.

Most participants received regular asthma treatment. Overall, 24 children (80.0%) were treated with inhaled corticosteroids (ICS), 10 (33.3%) with leukotriene receptor antagonists (LTRA), and 14 (46.7%) with long-acting beta-2 agonists (LABA).

Although 20 participants were classified at GINA treatment steps 3–4, only 14 were receiving LABA-containing regimens; other patients at these treatment steps received higher-dose ICS or ICS combined with an LTRA.

Treatment regimens included ICS alone in 7 children (23.3%), ICS combined with LABA in 7 (23.3%), ICS combined with LTRA in 3 (10.0%), and ICS combined with LABA and LTRA in 7 (23.3%). Six children had previously had an intermittent asthma but had experienced recent loss of asthma control, predominantly because of nocturnal and exercise-related symptoms, and were not receiving controller treatment at the time of the study.

During the 4 weeks preceding the study, the most common asthma-related symptoms were nocturnal symptoms, exertional chest tightness or pain, and occasional wheezing.

### 3.2. MII-pH Findings

Using the prespecified ≥1.2% threshold, 14/30 children (46.7%) were classified as MII-pH-positive. In a sensitivity analysis using the ≥1.3% threshold, 13/30 children (43.3%) were classified as MII-pH-positive. EIB occurred in 1/13 MII-pH-positive and 5/17 MII-pH-negative participants (OR = 0.20; Fisher’s exact *p* = 0.196), and the conclusion was unchanged.

There were no significant differences between MII-pH-positive (n = 14) and MII-pH-negative patients (n = 16) with respect to age, sex, absolute BMI, spirometric parameters, ACT score, treatment step, or exercise-related change in FEV_1_.

No significant associations were identified between the use of ICS, LABA, or LTRA and MII-pH parameters analyzed for either the complete recording or the separate pre-ECT and post-ECT periods.

### 3.3. Exercise-Induced Bronchoconstriction

EIB was diagnosed in 6 of 30 children (20.0%). The median decrease in FEV_1_ among children with EIB was 13.2% [IQR, 12.7–32.2].

Fourteen children (46.7%) reported mild or moderate exercise-related respiratory symptoms during the period preceding the study. Six of these children regularly participated in intensive sports. In some children with a negative spirometric ECT, mild transient coughing or isolated wheezing was noted during or shortly after exercise and resolved rapidly.

EIB occurred in 1/14 (7.1%) MII-pH-positive participants and in 5/16 (31.3%) MII-pH-negative participants (OR = 0.17; Fisher’s exact *p* = 0.175). No statistically significant association between MII-pH status and EIB was demonstrated.

Most 48 h MII-pH parameters did not differ significantly between children with and without EIB. An isolated difference was observed for total reflux exposure percentage time, which was lower in the EIB-positive group than in the EIB-negative group: 1.0 [0.7–1.6] versus 1.9 [1.1–2.5], respectively (*p* = 0.048). Given the small number of EIB-positive participants and the multiple comparisons performed, this finding was considered exploratory.

### 3.4. Comparison of MII-pH Parameters Before and After ECT

The median number of reflux episodes was higher during the post-ECT period than during the pre-ECT period: 43.0 [35.2–69.8] versus 33.5 [21.2–54.5], respectively (*p* = 0.006; Table 2).

The absolute number of reflux episodes was higher in the post-ECT segment in the original count-based analysis. However, in the 17 participants with retrievable segment-specific analyzable durations, the median reflux episode rate was 1.71 episodes/hour before ECT and 1.85 episodes/hour after ECT, with no significant paired difference (*p* = 0.782). No significant differences between the two recording periods were identified for mean acid clearance time, duration of the longest reflux episode, percentage time of impedance-detected acid reflux, non-acid reflux exposure percentage time, or total reflux exposure percentage time.

**Figure 1:**
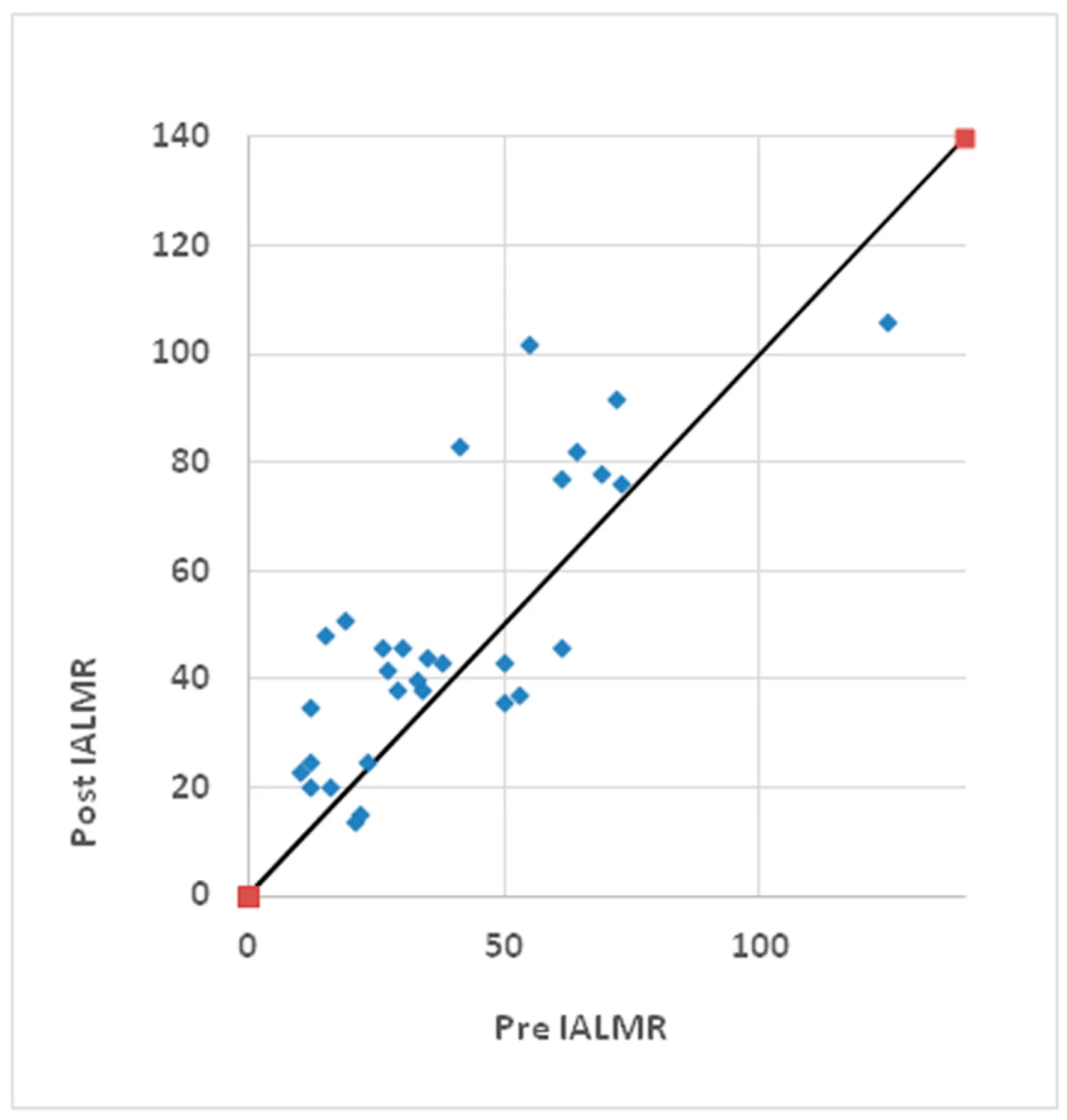
Scatter plot of impedance all liquid + mixed reflux (IALMR) before (pre IALMR) and after (post IALMR) the exercise challenge test. Each point represents one participant. The diagonal line represents the line of identity; points above the line indicate a higher number of reflux episodes after ECT, whereas points below the line indicate a lower number of reflux episodes after ECT.

Assessing the influence of ECT on MII/pH recording we found that in the 24 h recording following the physical effort raised the number of reflux episodes (pre/post ALMR, all liquid + mixed reflux, *p* = 0.006), as presented in Figure 1.

### 3.5. Comparison ACCORDING to MII-pH Status

During the post-ECT recording period, the duration of the longest reflux episode was greater in the MII-pH-positive group than in the MII-pH-negative group: 5.7 [3.7–8.2] versus 2.5 [1.5–4.7], respectively (*p* = 0.020).

Total reflux exposure percentage time was higher in the MII-pH-positive group both before ECT (2.4 [1.5–3.0] vs. 1.0 [0.6–1.4]; *p* < 0.001) and after ECT (2.0 [1.5–3.1] vs. 1.1 [0.5–1.7]; *p* = 0.010).

The number of reflux episodes was also higher in the MII-pH-positive group before ECT (45.5 [33.5–59.5] vs. 22.5 [14.2–38.0]; *p* = 0.013) and during the post-ECT period (48.5 [43.5–82.8] vs. 35.5 [22.2–46.0]; *p* = 0.003; Table 3).

These comparisons represent between-group differences and do not demonstrate a within-group increase in reflux parameters following ECT.

### 3.6. Nutritional Status and Reflux Parameters

Twenty-one participants (70.0%) had normal weight, 4 (13.3%) were overweight, and 5 (16.7%) had obesity. No participant met the World Health Organization criterion for thinness. The combined prevalence of overweight and obesity was 30.0%.

Abnormal acid exposure was identified in 7 of 9 participants (77.8%) with overweight or obesity and in 7 of 21 participants (33.3%) with normal weight.

Excess weight was associated with MII-pH-positive status (OR, 7.0; 95% CI, 1.14–42.97; *p* = 0.046).

Median percentage time of impedance-detected acid reflux for the complete recording was 1.7% [1.3–1.8] in participants with overweight or obesity and 0.9% [0.6–1.4] in participants with normal weight (*p* = 0.063).

BMI-for-age z-score was positively correlated with the percentage time of impedance-detected acid reflux (Spearman ρ = 0.41; *p* = 0.025).

These associations did not remain significant after Benjamini–Hochberg correction (q = 0.117 and q = 0.079, respectively) and should therefore be regarded as hypothesis-generating.

The total number of reflux episodes over the complete approximately 48 h recording did not differ significantly between participants with excess weight and those with normal weight: 84 [74–124] versus 68 [46–97], respectively (*p* = 0.353).

BMI-for-age z-score was not significantly correlated with the total number of reflux episodes (ρ = 0.22; *p* = 0.248).

### 3.7. Symptom Association

An SI above 50% and SAP above 95% were considered positive.

For the complete recording, belching SI was higher in the MII-pH-positive group than in the MII-pH-negative group (50% [33–73] vs. 0% [0–8.3], *p* = 0.002). Belching SAP was also higher in the MII-pH-positive group (84% [11.3–99.8] vs. 0% [0–0], *p* = 0.013). No participant had a positive respiratory SI or SAP for cough, wheezing, or dyspnea.

No participant had a positive respiratory SI or SAP for cough, wheezing, or dyspnea.

## 4. Discussion

In this prospective exploratory study of children with partially controlled or uncontrolled asthma and no typical reflux symptoms, abnormal acid exposure was identified in almost half of the cohort. We found no significant association between abnormal acid exposure and EIB. However, the number of reflux episodes was higher during the 24 h period following a standardized exercise challenge than during the preceding 24 h period. An additional exploratory finding was the association between excess body weight and MII-pH-positive status, with BMI-for-age z-score correlating positively with esophageal acid exposure.

Although associations between asthma, EIB, and GER have previously been described [39,40], studies combining MII-pH monitoring with a standardized exercise challenge in children with asthma remain limited. Jozkow et al. evaluated GER during physical activity in adults with confirmed GERD using conventional 24 h pH monitoring, which primarily assesses acid exposure [41]. MII-pH provides a broader assessment of reflux events, including bolus movement and esophageal clearance [42], and may therefore be particularly useful when typical reflux symptoms are absent.

In our cohort, abnormal acid exposure was present in 46.7% of participants. Previous studies using pH monitoring have reported widely varying rates of pathological acid exposure in children with asthma [14,43,44], probably reflecting differences in patient selection, diagnostic thresholds, and inclusion of symptomatic patients. Pediatric studies incorporating impedance measurements have also demonstrated a substantial burden of reflux in children with asthma [45,46,47,48]. Szaflarska-Popławska and Popławski reported abnormal reflux findings in 41.9% of children with asthma, a proportion similar to that observed in the present cohort [48].

We did not identify an association between MII-pH status and asthma control, treatment step, or the evaluated asthma medications. Other studies have likewise failed to demonstrate a consistent relationship between asthma control and reflux detected by MII-pH monitoring [48,49]. Asthma treatment was deliberately continued during the present study, and participants maintained their usual diet and daily routines. This pragmatic design was intended to assess reflux and bronchial responses under conditions resembling everyday clinical practice rather than after medication withdrawal.

Continuation of regular asthma treatment is particularly relevant to the interpretation of the ECT findings. Regular asthma treatment was continued during the study, including on the day of ECT. LABA and LTRA therapy may have attenuated exercise-related bronchoconstriction; therefore, the 20% prevalence of EIB observed in this cohort should not be interpreted as the untreated prevalence of EIB in children with inadequately controlled asthma. The relatively low prevalence of spirometrically confirmed EIB should also be interpreted in the context of pre-existing exercise-related symptoms and habitual physical activity. Fourteen participants reported mild or moderate exercise-related symptoms, whereas six were highly physically active and regularly participated in intensive sports. In such children, the standardized exercise challenge may have provided a relatively less provocative stimulus. Moreover, some participants with a negative spirometric ECT developed transient cough or isolated wheezing that did not meet the predefined ≥10% FEV_1_ criterion.

Only six participants met the diagnostic criterion for EIB, five of whom were MII-pH-negative and one MII-pH-positive. The direction of the observed association was opposite to the original hypothesis, although the estimate was highly imprecise because only six participants had EIB. The study was therefore underpowered to exclude a clinically relevant association.

The small number of EIB-positive participants substantially limited statistical power. Our findings therefore do not establish that GER and EIB are unrelated but indicate that no association was demonstrated in this cohort under usual asthma treatment conditions. The isolated finding of lower total reflux exposure percentage time in children with EIB should also be interpreted cautiously. This result arose from multiple comparisons and was based on only six EIB-positive participants; it should therefore be considered hypothesis-generating rather than evidence of a protective association between reflux and EIB. Although MII-pH monitoring continued during the exercise challenge, the study was not designed to analyze a predefined short peri-exercise reflux window. Therefore, an acute temporal relationship between reflux occurring during exercise and bronchoconstriction cannot be excluded.

The main finding relating ECT to GER was the higher number of reflux episodes during the 24 h period following ECT compared with the preceding 24 h period. Median reflux episode count increased from 33.5 to 43.0. This temporal pattern is consistent with previous observations that strenuous exercise may influence reflux burden. Herregods et al. demonstrated that running may provoke excessive reflux even in asymptomatic healthy adults [50]. Exercise-related reflux has been attributed to impaired esophageal motility, changes in lower esophageal sphincter function, increased transient lower esophageal sphincter relaxations, delayed gastric emptying, and mechanical factors associated with vigorous physical activity [21,50,51].

The higher absolute post-ECT episode count should be interpreted cautiously because the pre-ECT and post-ECT segments differed in analyzable duration. A rate-based sensitivity analysis in the subset with available segment-specific recording times did not confirm a higher reflux frequency after ECT. Therefore, the absolute count difference cannot be regarded as definitive evidence that exercise increased reflux frequency.

Moreover, the present findings should be interpreted in the context of the study design. The pre-ECT and post-ECT recording periods occurred in a fixed temporal sequence, and neither a control day without ECT nor randomization of exercise timing was included. Natural day-to-day variability in reflux, sleep, body position, diet, and spontaneous physical activity may therefore have contributed to the observed difference between the two recording periods. Although the ECT was performed at a standardized time 2 h after a hospital-provided breakfast and meal periods were excluded from the MII-pH analysis, these measures cannot distinguish an exercise-related effect from between-day variability. Therefore, a causal effect of exercise on reflux cannot be established.

During the post-ECT recording period, MII-pH-positive participants had longer maximal reflux episodes. The number of reflux episodes was also higher in the MII-pH-positive group during both the pre-ECT and post-ECT periods. These findings characterize differences between the predefined reflux groups and should not be interpreted as evidence of within-group deterioration in reflux or esophageal clearance following exercise.

The clinical significance of asymptomatic reflux in relation to respiratory symptoms remains uncertain. GER-related respiratory manifestations have been proposed to occur through microaspiration of gastric or duodenal contents or through vagally mediated pathways triggered by distal esophageal stimulation [13,52,53,54]. Experimental acid perfusion has been reported to increase bronchial hyperresponsiveness in patients with asthma [55], and non-acid reflux may contribute to cough or wheeze in selected patients [56]. Nevertheless, increased reflux burden in the present cohort was not associated with EIB or with positive respiratory SI or SAP. Thus, the reflux findings observed during the post-ECT recording period were not accompanied by clinically measurable post-exercise bronchoconstriction or a demonstrable temporal association with recorded respiratory symptoms in this cohort.

The association between nutritional status and reflux was an additional exploratory finding. Overweight or obesity was present in 30% of participants, and children with excess weight had approximately sevenfold higher odds of being classified as MII-pH-positive than children with normal weight. BMI-for-age z-score also correlated positively with acid exposure percentage time. In contrast, no significant association was identified between BMI-for-age z-score and the total number of reflux episodes.

Exploratory analyses suggested an association between excess body weight and MII-pH-positive status and a positive correlation between BMI-for-age z-score and acid exposure; however, these findings did not remain significant after adjustment for multiple testing. Increased intra-abdominal pressure, altered esophagogastric junction mechanics, and impaired reflux clearance may contribute to the relationship between obesity and acid reflux [24]. The interaction between asthma, obesity, and GER is likely complex, because obesity may independently increase respiratory symptoms and contribute to poor perceived asthma control while also promoting reflux and symptoms that overlap with asthma manifestations [23,25,57].

The nutritional status analysis was post hoc and exploratory and was not part of the original primary study objectives. Only nine participants were overweight or obese, and the confidence interval around the estimated odds ratio was wide. These findings should therefore be considered hypothesis-generating and require confirmation in larger pediatric cohorts.

No significant associations were identified between ICS, LABA, or LTRA use and MII-pH parameters. Although sympathomimetic medications have previously been proposed to reduce lower esophageal sphincter pressure [29,30], our findings do not suggest a strong association between usual asthma medication use and reflux burden in this cohort. However, the study was neither designed nor powered to assess medication effects. Treatment was not randomized, the treatment subgroups were small, and medication use was related to the clinical characteristics of asthma. Causal conclusions regarding asthma treatment and reflux therefore cannot be drawn.

This study has several limitations. First, the sample size was small, and only six children had EIB, limiting the ability to detect associations between reflux and bronchial responses. Second, no healthy control group or comparison group of children with well-controlled asthma was included. It therefore remains uncertain whether the difference in reflux episode count between the two recording periods was specific to children with inadequately controlled asthma or reflected a more general pattern. Although the within-subject design reduced interindividual variability by allowing each participant to serve as his or her own control, it did not replace an external control group. Therefore, the study cannot determine whether the observed reflux burden or temporal patterns are specific to children with inadequately controlled asthma.

Third, the pre-ECT and post-ECT periods were analyzed in a fixed sequence without a control day or randomized exercise timing. A causal effect of exercise on reflux cannot therefore be established. Fourth, regular asthma treatment was continued. Although this approach was consistent with the pragmatic objective of the study, LABA and LTRA treatment may have reduced the likelihood of a positive ECT.

Fifth, symptom association analyses depended on diary entries and use of the event marker. Incomplete symptom reporting may have resulted in underestimation of respiratory SI and SAP, particularly in older children [58]. Finally, the catheter did not include impedance channels or a pH sensor above the upper esophageal sphincter. Although proximal esophageal bolus movement could be observed, laryngopharyngeal reflux and microaspiration could not be reliably assessed.

Nominally significant secondary findings should be interpreted cautiously because of the number of exploratory comparisons performed.

Pre-ECT and post-ECT recording segments differed in analyzable duration. The duration-normalized sensitivity could be performed only in the subset with retrievable segment-specific recording times.

Furthermore, the standard ACT was used uniformly across the cohort, including participants younger than 12 years; because this instrument was originally validated in older children and adults, this represents an additional limitation.

Finally, the study is based on a prospectively collected dataset in years 2007–2011. Diagnostic approaches, pediatric MII-pH interpretation, and asthma management have evolved since the data were collected, which may limit direct applicability to contemporary clinical practice.

Despite these limitations, the study provides detailed 48 h MII-pH data obtained in children with inadequately controlled asthma undergoing a standardized exercise challenge while continuing their usual asthma treatment. The study therefore offers exploratory data on temporal differences in reflux burden before and after ECT under pragmatic clinical conditions.

Future studies should include larger cohorts, a non-exercise control period, and randomization of exercise timing. Predefined short-term post-exercise analysis windows may help distinguish an acute exercise-related effect from day-to-day variability in reflux. Inclusion of children with well-controlled asthma and healthy controls would further clarify whether observed differences in reflux burden are specific to inadequately controlled asthma. Studies using monitoring systems capable of assessing reflux above the upper esophageal sphincter may also help determine the potential respiratory relevance of proximal or laryngopharyngeal reflux.

## 5. Conclusions

Abnormal acid exposure was frequently identified by MII-pH monitoring in children with partially controlled or uncontrolled asthma despite the absence of typical reflux symptoms. No statistically significant association between abnormal acid exposure and EIB was demonstrated, although the small number of EIB-positive participants limits definitive conclusions.

The absolute number of reflux episodes was higher in the post-ECT segment; however, this difference was not confirmed after normalization to analyzable recording time in the subset with available segment-specific duration data. The findings therefore do not establish that exercise increases reflux frequency.

Associations involving nutritional status and other secondary reflux parameters should be considered exploratory and hypothesis-generating.

Larger controlled studies incorporating a non-exercise control period and predefined short-term post-exercise assessment windows are needed to clarify the clinical relevance of reflux following exercise in children with asthma.

## Figures and Tables

**Table 1:** Baseline characteristics of the study population (n = 30).

Feature	Value
**Age, years, mean ± SD**	12.7 ± 3.3
**Sex, n (%)**	
Male	18 (60.0)
Female	12 (40.0)
**BMI, kg/m^2^, median [IQR]**	20.1 [17.3–21.9]
**Nutritional status, n (%)**	
Normal weight	21 (70.0)
Overweight	4 (13.3)
Obesity	5 (16.7)
**Sensitization, n (%)**	
Indoor allergens	22 (73.3)
Outdoor allergens	14 (46.7)
**Concomitant allergic rhinitis, n (%)**	25 (83.3)
**Non-sedating H1 antihistamine use, n (%)**	12 (40.0)
**Asthma treatment, n (%)**	
ICS	24 (80.0)
LTRA	10 (33.3)
LABA	14 (46.7)
**Asthma control, n (%)**	
Partially controlled	4 (13.3)
Uncontrolled	26 (86.7)
**GINA treatment step, n (%)**	
1	6 (20.0)
2	4 (13.3)
3	17 (56.7)
4	3 (10.0)
**ACT score, median [IQR]**	18.0 [15.2–18.8]
**FEV_1,_ % predicted, median [IQR]**	98.5 [86.2–102.8]

Data are presented as mean ± standard deviation (SD), medians [interquartile ranges (IQR)], or number (percentage), as appropriate. BMI, body mass index; ICS, inhaled corticosteroids; LTRA, leukotriene receptor antagonists; LABA, long-acting β2 agonists; GINA, Global Initiative for Asthma; ACT, Asthma Control Test; FEV_1_, forced expiratory volume in 1 s.

**Table 2:** Parameters in MII-pH recording before and after the exercise challenge test (Pre– ECT and Post–ECT, respectively).

Parameter	Pre–ECT	Post–ECT	*p* Value
**Mean acid clearance time, s**	75.0 [47.2–97.5]	65.0 [44.0–98.2]	0.943
**Longest reflux episode, min**	3.9 [1.9–7.3]	3.8 [2.4–7.3]	0.863
**Impedance-detected acid reflux exposure, %**	0.9 [0.6–1.4]	1.1 [0.5–1.8]	0.482
**Non-acid reflux exposure, %**	0.5 [0.2–1.0]	0.4 [0.1–0.8]	0.677
**Total reflux exposure, %**	1.4 [1.0–2.2]	1.6 [1.1–2.5]	0.876
**No. of liquid and mixed reflux episodes**	33.5 [21.2–54.5]	43.0 [35.2–69.8]	**0.006**

Data are presented as medians [interquartile ranges]. ECT, exercise challenge test; MII-pH, multichannel intraluminal impedance–pH monitoring; Pre–ECT, recording period before the exercise challenge test; Post–ECT, recording period following completion of the exercise challenge test. Statistically significant *p* values (*p* < 0.05) are in bold. These comparisons were considered secondary/exploratory. Nominal *p* values are shown; Benjamini–Hochberg false discovery rate-adjusted q values are provided in Supplementary Table S1.

**Table 3:** Comparison of MII-pH parameters according to MII-pH status.

Parameter	MII-pH-Negative (n = 16)	MII-pH-Positive (n = 14)	*p* Value
**Pre-ECT mean acid clearance** **time, s**	57.0 [32.2–106.2]	88.0 [69.8–95.5]	0.244
**Post-ECT mean acid clearance time, s**	47.5 [33.5–80.5]	75.5 [65.0–115.5]	0.081
**Pre-ECT longest reflux episode, min**	2.1 [1.1–8.4]	4.7 [3.7–6.7]	0.109
**Post-ECT longest reflux episode, min**	2.5 [1.5–4.7]	5.7 [3.7–8.2]	**0.020**
**Pre-ECT impedance-detected acid reflux exposure, %**	0.6 [0.2–0.8]	1.6 [1.1–2.2]	NA
**Post-ECT impedance-detected acid reflux exposure, %**	0.6 [0.4–1.0]	1.7 [1.4–2.4]	NA
**Pre-ECT non-acid reflux** **exposure, %**	0.4 [0.2–0.6]	0.7 [0.4–1.1]	0.260
**Post-ECT non-acid reflux exposure, %**	0.4 [0.2–0.7]	0.4 [0.1–0.8]	0.851
**Pre-ECT total reflux exposure, %**	1.0 [0.6–1.4]	2.4 [1.5–3.0]	NA
**Post-ECT total reflux exposure, %**	1.1 [0.5–1.7]	2.0 [1.5–3.1]	NA
**Pre-ECT number of liquid and mixed reflux episodes**	22.5 [14.2–38.0]	45.5 [33.5–59.5]	**0.013**
**Post-ECT number of liquid and mixed reflux episodes**	35.5 [22.2–46.0]	48.5 [43.5–82.8]	**0.003**

Data are presented as medians [interquartile ranges]. MII-pH, multichannel intraluminal impedance–pH monitoring; Pre-ECT, 24 h recording period before the exercise challenge test; Post-ECT, 24 h recording period following completion of the exercise challenge test. MII-pH-positive status was defined by an acid exposure percentage time of ≥1.2% for the complete recording. NA, not applicable, between-group *p* values were not calculated for impedance-detected acid reflux exposure because MII-pH group status was defined using this parameter for the complete recording. Statistically significant *p* values (*p* < 0.05) are in bold. Between-group comparisons of impedance-detected acid exposure were not performed because this variable defined MII-pH status. Total reflux exposure includes the acid component and was therefore not used for independent inferential comparison between MII-pH groups. Nominal *p* values for the remaining exploratory comparisons are shown; Benjamini–Hochberg-adjusted q values are provided in Supplementary Table S1.

## Data Availability

Data are not openly available due to ethical/privacy restrictions as permission for data sharing was not provided by the participants or the Ethical Committee.

## Supplementary Materials

The following supporting information can be downloaded at: https://www.mdpi.com/article/10.3390/arm94050066/s1. Supplementary Table S1. False discovery rate adjustment of secondary and exploratory analyses.

## Abbreviations

The following abbreviations are used in this manuscript:

EIB	exercise-induced bronchoconstriction
ECT	exercise challenge test
FEV_1_	forced expiratory volume in 1 s
GER	gastroesophageal reflux
GERD	gastroesophageal reflux disease
MII-pH	multichannel intraluminal impedance and pH monitoring
GINA	Global Initiative for Asthma
ACT	Asthma Control Test
BMI	body mass index
SI	symptom index
SAP	symptom association probability
IQR	interquartile range
ICS	inhaled corticosteroids
LTRA	leukotriene receptor antagonists
LABA	long-acting beta-2 agonists
CI	confidence interval
OR	odds ratio
IALMR	impedance all liquid + mixed reflux
