# Supplementary material for: Gastroesophageal Reflux Burden Before and After Exercise Challenge in Children with Poorly Controlled Asthma: A 48-Hour MII-pH Study
Source: Adv Respir Med. 2026 Sep 16;94(5):66. doi: 10.3390/arm94050066 (PMC13600225; doi:10.3390/arm94050066)
Supplement: Supplementary file 1 [file arm-94-00066-s001.zip › arm-4484851-supplementary.pdf]

Supplementary Table S1. False discovery rate adjustment of secondary and exploratory analyses.

**Analysis**	**Nominal p value**	**BH-adjusted q**
Belching SI: MII-pH+ vs MII-pH−	.002	.033
Post-ECT reflux episode count: MII+ vs MII−	.003	.033
Pre- vs post-ECT absolute reflux episode count	.006	.044
Pre-ECT reflux episode count: MII+ vs MII−	.013	.057
Belching SAP: MII+ vs MII−	.013	.057
Post-ECT longest reflux episode: MII+ vs MII−	.020	.073
BMI z-score vs acid exposure	.025	.079
Excess weight vs MII-pH status	.046	.117
Total reflux exposure: EIB+ vs EIB−	.048	.117
Acid exposure: excess vs normal weight	.063	.139
Post mean acid clearance: MII+ vs MII−	.081	.162
Pre longest reflux episode: MII+ vs MII−	.109	.200
Pre mean acid clearance: MII+ vs MII−	.244	.381
BMI z-score vs total episode count	.248	.381
Pre non-acid exposure: MII+ vs MII−	.260	.381
Total episode count: excess vs normal weight	.353	.485
Pre/post acid reflux exposure	.482	.624

Analysis	Nominal p value	BH-adjusted q
Pre/post non-acid reflux exposure	.677	.827
Post non-acid exposure: MII+ vs MII−	.851	.918
Pre/post longest reflux episode	.863	.918
Pre/post total reflux exposure	.876	.918
Pre/post mean acid clearance	.943	.943

BH, Benjamini–Hochberg adjustment; SI, symptom index; MII-pH, multichannel intraluminal impedance–pH monitoring; ECT, exercise challenge test; SAP, symptom association probability; BMI, body mass index; EIB, exercise-induced bronchoconstriction.

Benjamini–Hochberg adjustment was applied jointly to the secondary and exploratory inferential tests retained in the revised manuscript. The prespecified primary association between MII-pH status and EIB and sensitivity analyses were not included in this multiplicity-adjustment family. Comparisons of acid exposure according to MII-pH status were not performed because acid exposure defined group status. Total reflux exposure comparisons according to MII-pH status were excluded from inferential testing because total exposure incorporates the acid component.
